# Does online high-volume hemodiafiltration offer greater efficiency and sustainability compared with high-flux hemodialysis? A detailed simulation analysis anchored in real-world data

**DOI:** 10.1093/ckj/sfae147

**Published:** 2024-05-10

**Authors:** Bernard Canaud, Alfred Gagel, Arne Peters, Andreas Maierhofer, Stefano Stuard

**Affiliations:** Nephrology Department, Montpellier University, School of Medicine, and Foundation Ch. Mion, AIDER-Santé, Montpellier, France; Global Research and Development, Fresenius Medical Care Deutschland GmbH, Care Enablement, Bad Homburg, Germany; Global Research and Development, Fresenius Medical Care Deutschland GmbH, Care Enablement, Bad Homburg, Germany; Global Research and Development, Fresenius Medical Care Deutschland GmbH, Care Enablement, Bad Homburg, Germany; Clinical & Therapeutic Governance, Fresenius Medical Care, Global Medical Office, EMEA Clinical & Therapeutic Governance, Bad Homburg, Germany

**Keywords:** end-stage kidney disease, green dialysis, renal replacement therapy, sustainable dialysis

## Abstract

Recent findings, including the CONVINCE (comparison of high-dose HDF with high-flux HD) study report, suggest the superiority of high-volume hemodiafiltration (HDF) over high-flux hemodialysis (HD) in improving patients’ outcomes. Despite positive patient outcomes, concerns have arisen about the potential negative environmental impact of high-volume HDF, as it may lead to increased water and dialysis fluid consumption and higher waste production. In this manuscript, we address the environmental impact of high-volume HDF, focusing on three key factors: water treatment consumption, dialysis fluid consumption, and solute efficiency markers of HD and HDF.

By optimizing HDF prescription through adjustments in operational capabilities, while keeping a high blood flow (i.e., >350 ml/min) such as reducing the Q_D_/Q_B_ ratio to 1.2 rather than 1.4 or 1.5 and incorporating automated ultrafiltration and substitution control, we demonstrate that HDF delivers a higher dialysis dose for small- and middle-molecule uremic compounds with the same dialysis fluid consumption, and at equal dialysis doses dialysis fluid consumption is reduced. This finding is supported by real-world data from 26 031 patients who underwent high-volume postdilution HDF at a reduced dialysis flow (430 mL/min) and achieved an effective _OCM_Kt/V of 1.70 (where “OCM” stands for online clearance measurement, “K” represents effective dialysis clearance and “V” denotes total body water measured by multifrequency bioimpedance). In addition, simulation modeling calculations, using blood extraction coefficient, dialysate saturation coefficient and solute clearances with urea (small molecular weight) and β2-microglobulin (middle molecular weight), consistently show the superiority of postdilution HDF to HD. This holds true even with a significant reduction in dialysis flow down to 430 mL/min, reflecting Q_D_/Q_B_ ratio of 1.2. Postdilution HDF generates high ultrafiltrate flow (up to 35% of blood flow), delivering saturated ultrafiltrate to the lower solute concentration containing effluent dialysate, thus enhancing solute clearance which opens the way to reduce the dialysis flow. In conclusion, our analysis, combining simulation and real-world data, suggests that postdilution HDF could be a more environmentally friendly treatment option compared with conventional HD. Additionally, automated user-friendly functions that minimize dialysis fluid use can further strengthen this environmental benefit while enhancing efficiency.

## WHAT DOES SUSTAINABILITY MEAN IN HEMODIALYSIS AND HEMODIAFILTRATION SETTING?

Sustainability in dialysis is a broad theme. One focal point is the ability to provide adequate renal replacement therapy while minimizing negative impacts on the environment, conserving resources and ensuring long-term economic viability [1, 2]. It encompasses the following aspects: the efficient use of water, energy and consumables, as well as reducing waste and CO_2_ emissions associated with the treatment process. Sustainable hemodialysis (HD) aims to strike a balance between the needs of chronic kidney disease patients, the impact on the environment and economic burden. It seeks to ensure that renal replacement therapy can be sustained for future generations without depleting resources or harming the planet. It is widely acknowledged that, in general, renal replacement therapy through dialysis is not an eco-friendly treatment [3–6]. Generally, a higher consumption of resources is associated with higher costs for generation and disposal of waste. Therefore, “green dialysis” and cost reduction go hand in hand [6, 7].

Recent findings confirm that high-volume hemodiafiltration (HDF) tends to be superior to high flux HD in improving outcomes for dialysis patients [8–11]. This conclusion is based on a significant reduction in the relative risk of all-cause mortality by 23%, as demonstrated in a large pragmatic randomized interventional trial known as the CONVINCE (comparison of high-dose HDF with high-flux HD) study [12]. Although still debatable [13–16], these results reinforce the findings of previous studies, both observational and interventional, which have consistently linked HDF with a reduced risk of mortality, especially related to cardiovascular causes [8, 9]. Notably, this mortality reduction was achieved through the prescription of a high substitution volume, which serves as a surrogate for the convective dialytic dose. This volume is set at a minimum of 23 L per session for postdilution HDF [8, 9, 17, 18].

However, despite the positive patient outcomes, the dose dependency has raised concerns about the potential negative environmental impact of high-volume HDF, as it may lead to increased water and dialysis fluid consumption and higher waste production. As such, Shroff *et al*. and the EUDIAL Working Group have raised a thought-provoking question regarding the sustainability and environmental impact of high-volume HDF [13]. This is due to the perception that substitution volume, so called substitute, in HDF must be spent on top of the dialysate volume prescribed in HD.

In this manuscript, to address the environmental question concerning high-volume HDF, we undertake an in-depth exploration of specific resource consumption and clinical performance, relying on a combination of precise simulations and real-world data. Our investigation focuses on comparing high-volume HDF with high-flux HD, particularly within the framework of conventional treatment schedules, which typically involve 4-h sessions three times a week.

## WHAT COMPONENTS NEED TO BE CONSIDERED IN THIS ASSESSMENT?

In the context of extracorporeal blood purification treatments like HD and HDF, various components are essential to ensure treatment safety and efficiency. These components can be categorized into three main pillars.

(i) A dedicated water treatment system that produces high-quality water designed for use in high flux HD and HDF [19].
(ii) The bioexchanger device, commonly referred to as a hemodialyzer or hemodiafilter, depending on its design. It plays a crucial role in facilitating blood filtration and purification [20].
(iii) The HD/HDF monitor, responsible for producing dialysis fluid and for the transport of blood through the extracorporeal blood circuit (EBC), maintaining the safety of the EBC and monitoring the patient throughout the treatment [21].

Additionally, consumable products are required for the procedure, including tubing sets and connection materials (such as gauzes, tapes, dressings and disinfectants), as well as the necessary electricity and labor, all of which also have environmental impacts. On the other hand, waste products are generated as byproducts of the treatment, consisting of unused water, dialysate enriched with uremic compounds, and waste from disposable plastic consumables. All these components have a serious environmental impact. Here we focus solely on the total water consumption, accompanied by dialysate concentrate and energy expenditures. Less water spent for dialysis means also less amount of wastewater generated.

## HOW DOES HIGH VOLUME HEMODIAFILTRATION COMPARE WITH HIGH FLUX HEMODIALYSIS FROM AN ANALYTICAL PERSPECTIVE?

In our current analysis, we will focus on water and dialysate consumption in relation to the patient outcomes achieved, which results from clinical performance of the system. Performance in this context will be evaluated using efficiency indicators commonly employed in HD. To simplify our approach, all calculations and simulations will be based on a single dialysis session. It is worth noting that most patients typically undergo three sessions per week, each lasting 4 h, amounting to 12 h per week and 156 sessions per year.

It is also essential to acknowledge that the consumption of dialysis fluid is heavily influenced by the various technical features available in the dialysis monitor used. Not all HDF monitors offer specific functionalities, such as automatically adjusting dialysis flow to match blood flow, considering user settings [namely the Autoflow function in Fresenius Medical Care (FMC) branding technology], or adapting substitution flow rates to account for changes in transmembrane pressure and dialyzer viscosity (namely Autosub plus, a substitution fluid control system in FMC branding technology) [22]. Therefore, it is crucial to exercise caution when interpreting our analysis, as generalizations may not be applicable unless the appropriate HDF monitor is employed, one that supports such specific functionalities. Otherwise, the user must set the dialysate or substitution flow rates manually to optimal values.

In this analytical description, we have selected three key factors for consideration: water treatment consumption, dialysis fluid consumption, and solute efficiency markers of HD and HDF.

### Production and consumption of water for dialysis

The water treatment systems for HD and HDF depend on a complex series of devices to effectively remove all solute compounds from tap water that includes both organic and inorganic substances. This water treatment system is common to both modalities. Without delving into the intricate details of the water treatment process, it is important to recognize that it comprises four stages.

(i) Pretreatment: this stage involves the clarification and the removal of most organic substances (e.g. pesticides, insecticides, agricultural) and non-organic compounds (e.g. metals, metalloids, electrolytes like calcium, magnesium).
(ii) Primary treatment: this comprises a single reverse osmosis (RO) stage, effectively removing most of the remaining electrolytes and contaminants, including microbial byproducts such as endotoxins.
(iii) Secondary treatment: this represents an additional step in water purification, achieved by either a second RO unit in series or adding a deionizer in series. This last stage is focused on elevating water purity levels and guaranteeing the complete removal of any remaining contaminants.
(iv) Tertiary treatment: to further enhance the purification process, involving sterilizing ultrafiltration, this is recommended to eliminate microorganisms and microbial-derived products downstream of the RO system before the water is channeled into the distribution loop. Subsequently, the purified water is distributed through a recirculating loop that supplies the dialysis machines.

It is well established that ultrapure water is essential for the treatment of HD patients, improving system biocompatibility and reducing inflammation. Highly purified water is a common requirement for both low- and high-flux HD, and HDF, not specific to the latter [23]. In this context, it is noteworthy to compare the water consumption of HD/HDF machines with the water production from water treatment system. On one hand, recent technical reports suggest that each dialysis session requires the production of approximately 320 L of ultrapure water [24, 25] when an optimal water treatment system is employed. On the other hand, the water consumption of HD machines during a 4-h treatment, depending on the dialysis fluid production, is 144 L when set at 600 mL/min, or 120 L when set at 500 mL/min. The significant disparity in water consumption in this process is attributed to wastage, including rinsing, flushing, detassing, regeneration processes and rejection from the RO system. A recent study found that dialysis machines in a dialysis unit utilize only 45% of the total treated water produced per day, with the remaining 65% being discarded in the hygienic and flushing procedures necessary to maintain the operability of the RO system [24]. Notably, the volume of water used is consistent across both HD and HDF modalities.

To ensure the microbiological safety of dialysis fluid at the point of care within the dialysis treatment, a final sterilizing ultrafilter is necessary in the dialysis fluid pathway [26, 27]. This guarantees the delivery of ultrapure dialysis fluid to the dialyzer. In online HDF, a second sterilizing ultrafilter is required for redundancy reasons for the substitution fluid to maintain first-failure safety sterility during its infusion into the patient's bloodstream, whether in the venous segment (postdilution mode) or the arterial segment (predilution mode). In both cases, these sterilizing ultrafilters are integral components of the dialysis monitor and benefit from disinfection and rinsing cycles.

From this perspective, the main difference in the dialysis fluid pathways between HDF and HD machines is the number of dialysate sterilizing filters used. HD utilizes a single dialysate sterilizing filter, whereas HDF requires two. For safety reasons, these sterilizing ultrafilters are periodically replaced, typically after 3 months of use or after 100 treatments. In the case of online HDF, this translates to eight dialysis fluid filters per year, compared with four in HD.

The first sterilizing filter is regularly backwashed, using 0.15 L per 30 L of generated ultra-pure dialysis fluid. The second sterilizing filter needs no backwash due to the pure input conditions. Generating ultrapure dialysate and sterile substitution fluid consumes no noteworthy additional water inside the dialysis monitor.

### Dialysis fluid production and consumption

Nevertheless, the advanced capabilities of online HDF machines allow for the optimization of water consumption by aligning dialysis flow with blood flow (Q_D_/Q_B_) or setting it at a fixed ratio to optimize dialysis fluid usage [28]. In essence, online HDF has the potential to reduce both dialysate and water consumption while maintaining identical or even increasing clinical performance, as explained in the following paragraph.

Dialysis machines are typically configured to produce 300–800 mL/min of dialysis fluid, depending on the user's prescription needs and settings.

**Figure 1:**
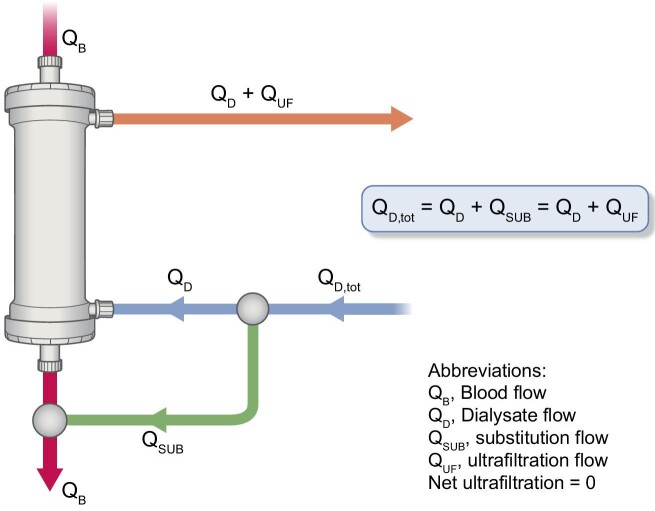
Schematic description of the online HDF system (postdilution), highlighting the fluid balancing system (Q_D_ inlet/outlet), which is ensured by the ultrafiltration control on the HD monitor. As not reflected in this cartoon, it must be noted that the substitution and dialysate flow passing through the dialyzer can be managed independently by the HDF monitor, as is the case with FMC machines.

In online HDF, as described in Fig. 1, a fraction of the total dialysis fluid (Q_D_,_tot_) is directed into the patient's bloodstream, either in the venous segment (postdilution mode) or the arterial segment (predilution mode). This redirected flow (Q_SUB_) typically constitutes 15%–35% of the total dialysis fluid (Q_D_,_tot_). The ultrafiltration control of the HD monitor compensates for this, by increasing the ultrafiltration rate drawn from the patient's plasma water (Q_UF_), thus in the absence of net ultrafiltration making Q_SUB_ and Q_UF_ equal. This configuration setting ensures precise fluid balance between the inlet and outlet dialysate flow of the dialyzer.

It is important to note that the total dialysate flow production (Q_D_,_tot_) of all HDF monitors is set according to user prescription. However, depending on the specific features of the HDF monitor, the substitution flow (Q_SUB_) and dialysate flow through the dialyzer (Q_D_) may be managed independently or linked to each other. This feature is crucial to consider as it may impact solute clearance performances as well as the amount of total spent dialysate.

**Figure 2:**
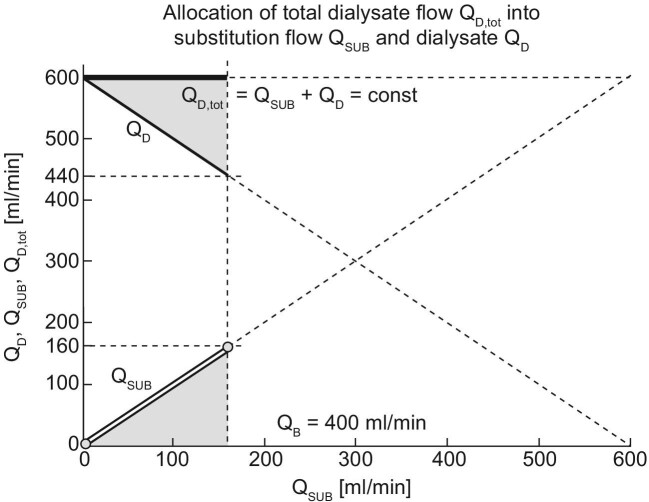
The allocation of the total dialysate flow (Q_D, tot_) into substitution (Q_Sub_) and dialysate (Q_D_) flow, and its distribution when adjusting the Q_D_/Q_B_ ratio either manually or automatically with an automated flow adjustment set by the user. This figure serves as the basis for the simulation depicted in Fig. 3.

In the online HDF machine, the impact of changing the ratio of substitution flow (Q_SUB_) to the dialysate flow (Q_D_) through the dialyzer is briefly outlined in the following diagram in Fig. 2, while the total dialysate flow (Q_D_,_tot_) remains constant.

As previously mentioned, compensating for substitution flow diversion (Q_SUB_) involves an equivalent reduction in the dialysis flow through the dialyzer (Q_D_) to maintain a constant total dialysate flow. In this configuration, the substitution flow (represented by the hollow line in Fig. 2, Q_SUB_) acts as a surrogate for ultrafiltration flow (Q_UF_), ensuring that fluid balance is maintained between the inlet and outlet dialysate pathways of the dialyzer. Interestingly, this diagram also easily demonstrates that with online HDF, an increase in substitution flow (Q_SUB_) results in an increase in ultrafiltration flow (Q_UF_). While the solute concentration of ultrafiltration flow Q_UF_ can approach that of the blood concentration, the concentration in the dialysate used for diffusive exchange will always be lower than the blood concentration. Consequently, in HDF, relatively poorly saturated dialysate is replaced by more saturated ultrafiltrate. In other words, for the same total dialysate flow produced, the fraction of more saturated dialysate increases with increasing substitution flow.

The consumption of dialysate and water in online HDF is defined by the settings and functionalities of the HDF machines. Two main methods for managing dialysis flow affect this consumption.

- In the first HDF machine setting, substitution flow is drawn from the total dialysis fluid flow without compensation. This results in a reduction of the dialysis fluid flowing through the dialyzer by the same amount.
- In the second setting, dialysis fluid flow and substitution flow are independently set, ensuring that the dialysis fluid passing through the dialyzer is unaffected by the substitution flow. Thus, substitution increases total dialysate flow. This is currently the most widespread modality in clinical practice.

Some HDF machines provide the option to align the dialysis fluid flow automatically with the blood flow (Q_D_/Q_B_) to optimize solute saturation and dialysate consumption. In all cases, it is crucial to maximize blood flow (QB) (ideally >350 ml/min) in order to optimize solute clearance and blood volume processed within a 4 hour treatment. The dialysate-to-blood flow ratio may be set, for example, to 1.5 in HD versus 1.2 in online HDF. This feature is built into FMC HDF machines [22].

Therefore, when addressing concerns about dialysis fluid and water consumption in the context of HDF, it is crucial to precisely match these setting considerations with the specific type of HDF machines used.

**Figure 3:**
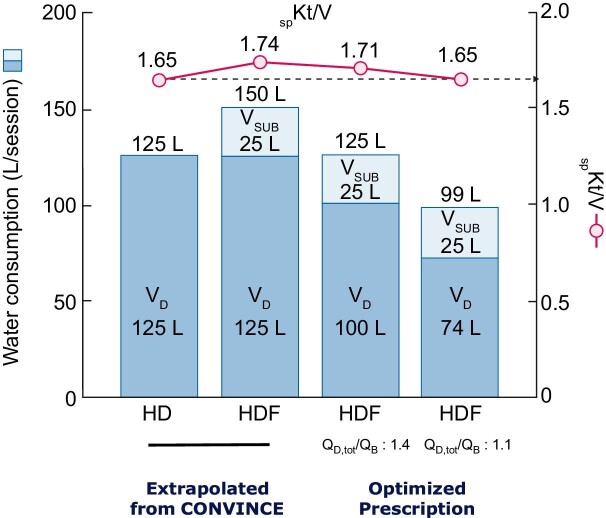
The estimated water consumption in CONVINCE based on the prescription (bar 1: HD 125 L; bar 2: HDF 150 L) and compares it with an optimized HDF prescription using automated dialysate flow adjustment (AutoFlow^®^) and automated substitution adjustment (AutoSub^®^) modes. This optimization aims to (a) spend the same amount of water (bar 3: VD, tot as in CONVINCE, with a Q_D_/Q_B_ ratio set at 1.4) and (b) achieve the same treatment efficiency (urea Kt/V 1.65) (bar 4: spKt/V as in CONVINCE setting witn a Q_D_/Q_B_ ratio set at 1.2) compared with CONVINCE HD-arm (bar 1).

As an example, let us examine the CONVINCE prescription as schematically presented in the Fig. 3 [12]. Based on the presumed average CONVINCE prescription, we estimated water consumption corresponding to delivered Kt/V values of 1.65 in HD and 1.75 in HDF [29, 30], observed in the study and based on average blood flows (Q_B_) and substitution volumes (V_SUB_) shown in Fig 3.

(i) Assuming an average Q_D_/Q_B_ ratio of 1.4, reflecting CONVINCE practices in the HD arm and in the HDF arm, yields a dialysate flow (Q_D_) of 520 mL/min. The dialysis fluid production would be (0.520 × 240)  L or 125 L of water per session with the treatment time of 240 min.
(ii) In HDF, assuming a total dialysate flow (total Q_D_) of 625 mL/min, consisting of a dialysate flow (Q_D_) of 520 mL/min and 105 mL/min (= 25.2 L/240 min) diverted as substitution flow (Q_SUB_), the total dialysis fluid production and water consumption would be 150 L per session (0.625 × 240), including 25.2 L used as substitution.
(iii) Now, consider an optimized approach to prescribing HDF, where the user configures the HDF machine with the total dialysate flow rate of Q_D, tot_ = 520 mL/min (Q_D, tot_/Q_B_ ratio of 1.4) equal to the dialysate flow in HD, but keeps the substitution rate at Q_SUB_ = 105 mL/min. The dialysate flowing dialyzer would be Q_D_ = 415 mL/min. The postdilution HDF treatment would use 0.52 × 240 = 125 L as in HD, but the dialysis dose would be _sp_Kt/V = 1.71, which is more efficient than in HD with _sp_Kt/V = 1.65, spending the same dialysate volume.
(iv) A further optimized approach to prescribing HDF spending less water than in HD is where the user configures the HDF machine with a Q_D, tot_/Q_B_ ratio of 1.1 (Q_D, tot_ 410 mL/min) while maintaining the same substitution flow (Q_SUB_ 105 mL/min; V_SUB_ 25.2 L). This configuration will provide comparable clearance of small solutes to HD (Kt/V of 1.65) but resulting in a total dialysis fluid production and water consumption equivalent to 99 L per session. In simpler terms, using a lower Q_D, tot_/Q_B_ ratio of 1.1 in HDF leads to reduced dialysis fluid production and water consumption while maintaining urea clearance performance. This result is facilitated by the fact that the saturation of solute within the effluent dialysate increases with the addition of fully saturated ultrafiltrate, as opposed to the low dialysate saturation achieved by diffusion in HD.

In simpler terms, while using a high blood flow (i.e., >350 ml/min) but optimizing the prescription of HDF through adjustments in operational capabilities of the HDF monitor, including reducing the Q_D_/Q_B_ ratio and incorporating automated ultrafiltration and substitution control, HDF proves to be a more sustainable modality than conventional HD.

To illustrate the benefits of automated dialysis for blood flow adjustment (Q_D_/Q_B_) from a dialysate-saving perspective, we utilized data from NephroCare clinics collected throughout the entire year of 2023, encompassing 21 European countries. In this cross-sectional analysis, we gathered information from 26 031 prevalent patients, with a dry weight of 74.8 ± 7.5 kg and total body water of 35.7 ± 7.7 L measured by bioimpedance. These patients were treated through arteriovenous fistula/graft in 83% of cases, with a treatment time of 242 ± 49 min per session. All patients received postdilution HDF, and we assessed dialysis performance at a dialysis to blood flow ratio Q_D_/Q_B_ set at 1.2. The further treatment prescriptions were as follows: blood flow set at 360 mL/min, and total dialysis flow at 434±72 mL/min. Additionally, the substitution volume was 25.7 ± 4.8 L, and the ultrafiltration volume was 27.4 ± 4.9 L. The dialysis dose (_OCM_Kt/V) was 1.70 ± 0.34 measured at each session, where “OCM” stands for online clearance measurement, “K” represents effective dialysis clearance and “V” denotes total body water measured by multifrequency bioimpedance. In other words, the findings of this real-world study confirm the previously calculated projected values. This prescription has been implemented in the network as a best practice for HDF over the last 10 years. It has proven to be both safe and efficient, and it also helps to prevent waste of dialysis fluid and water. As shown in Fig. 3, each high-volume HDF session can save up to 25 L of dialysis fluid, which translates to 3900 L per year per patient. This represents a substantial annual water saving, considering that 26 051 patients received high-volume HDF as their regular treatment in this network.

### Solute clearance efficiency markers

**Figure 4:**
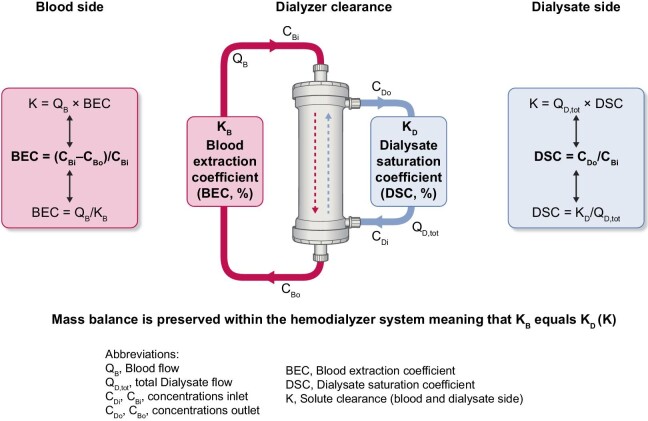
Schematic representation of indicators (BEC and DSC) and clearance efficiency (K) for solute removal.

To compare the effect of postdilution HDF versus high-flux HD, we have considered three indicators of solute removal performance: (i) blood extraction coefficient (BEC) as reflected by the ratio K/Q_B_, (ii) instantaneous solute clearances (K) and (iii) dialysate saturation coefficient (DSC), i.e. the ratio K/Q_D,tot_ [31–35]. We have selected two solute markers to illustrate our purpose, reflecting compounds of low molecular weight (urea, 60 Da) and middle molecular weight [β2-microglobulin (β2M), 11.8 kDa]. Fig. 4 provides a schematic representation of these indicators (BEC and DSC) and the clearance efficiency for solute removal of interest, as well as their mass balance link within the dialyzer.

To perform these calculations and simulations, the typical ratio between K_0_A β2M and K_0_A urea was derived from a data set coming from 1200 measurements collected in 65 HD patients, providing the relation K_0_A β2M = K_0_A urea/17.2. Using this relation, simulations with different flow rate settings were simulated in a calculator tool (Clearance Calculation Tool V2.0, FMC). These simulations are presented in the next paragraph.

**Figure 5:**
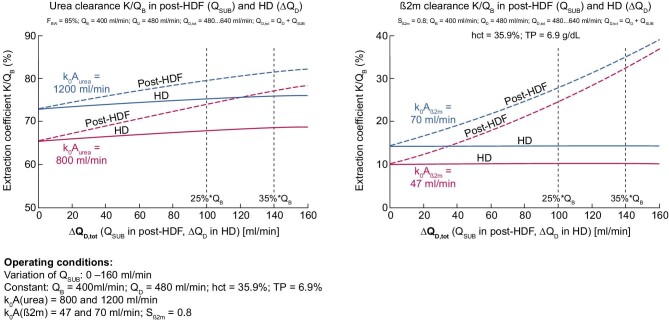
The BEC calculated for urea (left) and β2M (right), utilizing distinct mass transfer coefficients: K0A (β2M) = K0A (urea)/17.2.

**Figure 6:**
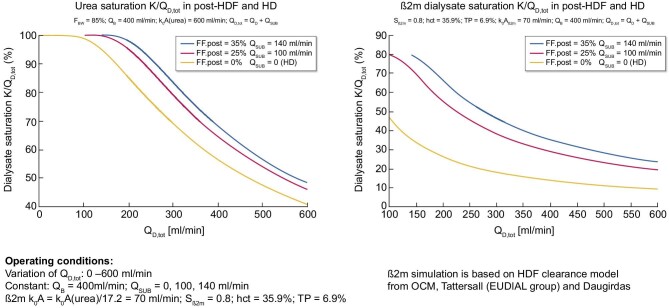
Illustration of the DSC calculated for urea (left) and β2M (right), employing distinct mass transfer coefficients: K0A (β2M) = K0A (urea)/17.2.

**Figure 7:**
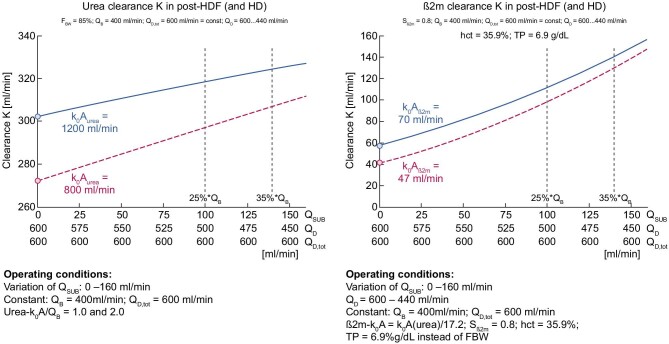
The clearance for urea (left) and β2M (right), ranging from high-flux HD (circles, starting point) to postdilution HDF (lines, right side), with an increasing filtration fraction (25% to 35%) while maintaining a constant total dialysate flow (600 mL/min).

(i) The first set of calculations is based on the BEC, which reflects the proportion of blood completely cleansed of a specific solute. BEC represents the ratio K/Q_B_ = (C_Bi_ – C_Bo_)/C_Bi_ for any solute, where K is the clearance, C_Bi_ and C_Bo_ stand for the arterial and venous concentration of the solute. This applies to urea and β2M, assuming a constant blood flow without net ultrafiltration (fluid removal). Simulations of BEC for HD and postdilution HDF (Y-axis) as a function of variable filtration fraction (X-axis) with currently available Polysulphone dialyzers (K_0_A urea = 1200 and 800 mL/min, and K_0_A β2M = 70 and 47 mL/min or K_0_A β2M = K_0_A urea/17.2) have been plotted in two graphs in Fig. 5 using a fixed blood flow of 400 mL/min. Various families of graphs have been generated for urea (Fig. 5, left) and for β2M (Fig. 5, right). For this representation, K_0_A to blood flow (Q_B_) ratio has been matched to reflect usual clinical practices where K_0_A/Q_B_ is between 1 and 3. As shown, the BEC in HD represents a nearly horizontal baseline with increasing dialysate flow rate ΔQ_D_, starting from Q_D_ = 480 mL/min. The BEC in postdilution HDF, with Q_D_ = 480 mL/min, is increasing with filtration rate Q_SUB_, with a significantly steeper slope for β2M than for urea. These families of graphs clearly underscore the superiority of postdilution HDF in BEC, more markedly with β2M than with urea.
(ii) The second set of calculations is based on the DSC, which serves as an indicator of how efficiently the spent dialysate is utilized, as presented in Fig. 6. DSC represents the ratio K/Q_D, tot_ = C_Do_/C_Bi_ and is valid for any solute, where K is the clearance, C_Do_ is the solute concentration in the dialysate outlet and C_Bi_ stands for the inlet concentration of the solute. This relationship applies to both urea and β2M, with no ultrafiltration and constant blood flow. Various families of graphs have been generated, plotting DSC on the Y-axis versus total dialysate flow (Q_D, tot_), considering a constant blood flow of 400 mL/min. DSC for urea is presented on the left side, and for β2M on the right side. As shown, postdilution HDF provides superior DSC across all dialysate flows compared with HD. Interestingly, DSC tends to increase slowly with reduced dialysate flow, reaching asymptotically (corresponding to pure hemofiltration) full dialysate saturation for urea (DSC = 1.0) and for β2M (DSC ≈ 0.8). These maximum saturation values may be used as a surrogate for the sieving coefficient of the membrane used in HD or HDF.
(iii) The third set of calculations is based on instantaneous solute clearances (urea and β2M), HD and postdilution HDF, as presented in Fig. 7. In this scenario, the solute K for both urea and β2M is graphed against various dialysis flows, assuming a constant total dialysate flow of 600 mL/min, a constant blood flow of 400 mL/min and no net ultrafiltration. Intriguingly, solute clearances for urea and β2M demonstrate an increase as dialysate flow through the dialyzer decreases, and substitution flow rises across all instances, clearances attained in postdilution HDF surpass those in HD mode (Q_SUB_ = 0, circles at the beginning of the graphs). Here, a filtration fraction of 25% might signify the postdilution HDF in a manual setting, while 35% may denote the HDF setting, when consistently administered in an automated ultrafiltration-controlled mode.

As clearly shown in this simulation, postdilution HDF is superior to HD in all cases for the two markers selected, reflecting low- (urea) and middle-sized (β2M) molecular weight solutes. This finding holds true despite a significant reduction of dialysis flow through the dialyzer down to 430 mL/min. As illustrated by the trend values in the DSC simulation, postdilution HDF, which generates high ultrafiltrate flow (up to 35% of blood flow), delivers highly saturated ultrafiltrate to the dialysate. This phenomenon increases the saturation of the dialysate and enhances solute clearance even while reducing the dialysate flow in the dialyzer pathway.

## HOW DOES HIGH VOLUME HEMODIAFILTRATION COMPARE TO HIGH FLUX HEMODIALYSIS FROM AN INTEGRATED PERSPECTIVE?

As quantified and emphasized in the preceding simulation sections, online postdilution HDF demonstrates superior efficiency in removing solutes across the entire molecular spectrum, ranging from low- to middle-sized molecular weight compounds. This finding is consistent with all experimental [36] and clinical performance reports [37–43]. Additionally, it possesses the capacity to reduce both water and dialysis fluid consumption while achieving the same low molecular clearance as HD and enhancing middle molecular clearances as recently confirmed in the CONVINCE study [12]. This remarkable action is attributed to the basic physical laws governing solute transport [29, 33].

Filtration, like that observed in healthy kidneys, is a much more efficient process than diffusion for removing solutes. This is because filtration can remove solutes regardless of their molecular weight, if they are smaller than the membrane's pores. However, achieving higher solute clearances with convection relies on adding it to the diffusive component, which can increase dialysate concentration (saturation) while reducing dialysis fluid consumption. This concept led to the development of postdilution hemofiltration, which aims to ensure full saturation of solutes within the ultrafiltrate [44–47]. It was soon recognized that the required convective volume in pure hemofiltration was too high to match the performance of HD on small molecular weight solutes. Therefore, HDF was developed as a hybrid modality. It retains the removal of small solutes while significantly enhancing the removal of middle and large ones, all while mitigating dialysis fluid consumption. Optimized HDF utilizes the automated functionalities of a HDF monitor. As previously mentioned, both dialysis fluid consumption and ultrafiltration flow can be automatically adjusted by online HDF machines using specific algorithms. In this optimized approach, HDF may adjust dialysis flow to blood flow and ultrafiltration flow by maximizing the filtration fraction. This leads to an enrichment of the dialysate with uremic solutes and increased dialysate saturation. This is why postdilution HDF stands out as significantly more efficient than high-flux HD, with a substantial reduction in dialysis fluid and water consumption. Building on this concept, the saturation ratio of the dialysate becomes a key marker for evaluating “green dialysis” efficiency. It best indicates the superiority of HDF or slow-flow HD system [31], [48], commonly used in slow daily dialysis treatment schedules, or with a multipass dialysate system [49]. As mentioned earlier, HDF is a more attractive option due to its potentially lower water consumption. Advanced HDF management technology in some monitors allows for even further reductions without sacrificing performance.

An additional cautionary note must be emphasized regarding the generalizability of our findings. These results were obtained from a European population with specific anthropometric characteristics, including body weight, body mass index, ethnicity and patient profile relying on prespecified operating conditions. Hence, it is imperative to demonstrate that in populations with different characteristics, such as higher body weight or body mass index, or varying medical profiles, or different practice patterns (i.e., Q_B_, tHD, substitution mode) the reduction in dialysis flow in favor of increased convective flow does not compromise or diminish diffusive dialysis clearances. Such aspects deserve further study for real-world validation.

## WHAT IS THE CONCLUSION?

**Figure 8:**
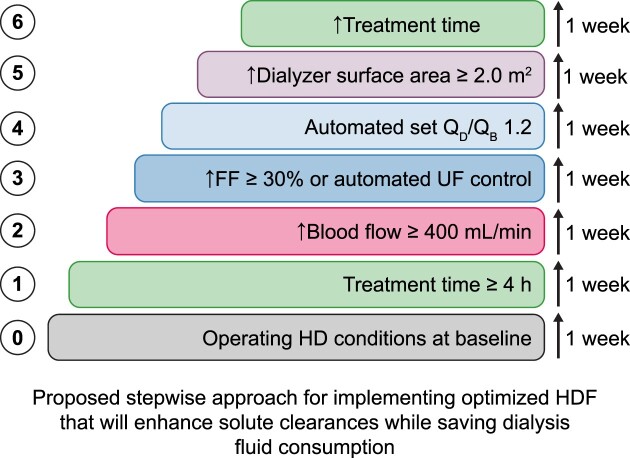
The proposed stepwise approach for implementing optimized HDF that will enhance solute clearances while saving dialysis fluid consumption.

For those seeking environmentally friendly options in renal replacement therapy, optimized postdilution HDF might be a strong contender compared with high-flux HD. This benefit is particularly significant when the treatment is prescribed using automated functions that manage dialysis fluid use efficiently. In such cases, HDF demonstrates a clear advantage in removing solutes across a wider range of molecular weights. As schematically presented in Fig. 8, a stepwise approach has been summarized to achieve an optimized prescription in HDF. Additionally, it offers the potential to reduce both water and dialysate use. This is achieved by subtly increasing the dialysate's solutes concentration, which is directly linked to the filtration fraction and ultrafiltration rate achieved during HDF session. In this context, postdilution HDF proves to be more efficient than high-efficiency high-flux HD. Interestingly, a larger convective volume, indicated by a higher filtration fraction, enhances the efficiency of HDF while maintaining total water consumption, thereby improving the water conservation capability of high-volume HDF.

This simulation study, based on rigorous simulations and real-world data, suggests that postdilution HDF may be environmentally preferable treatment option. This finding might seem surprising at first, but it is supported by the data. Notably, optimal prescription and easy implementation through automated functions, which minimize dialysis fluid and water use, will further enhance this environmental benefit.

## Data Availability

This is based on a modeling and simulation approach. Therefore data may be shared on a reasonable basis reflecting modele and calculation approach.
